# Systemic lupus erythematosus complicated by Crohn’s disease: a case report and literature review

**DOI:** 10.1186/1471-230X-12-174

**Published:** 2012-12-05

**Authors:** Hiroyuki Yamashita, Yo Ueda, Hoshimi Kawaguchi, Akitake Suzuki, Yuko Takahashi, Hiroshi Kaneko, Toshikazu Kano, Akio Mimori

**Affiliations:** 1 Division of Rheumatic Diseases, National Center for Global Health and Medicine, 1-21-1 Toyama, Shinjuku, Tokyo, 162-8655, Japan

**Keywords:** Systemic lupus erythematosus (SLE), Crohn’s disease (CD), Longitudinal ulcer, Aphthous ulcers, Cobblestone-like inflammatory polyps, Non-caseating granuloma, Vasculitis, Infliximab

## Abstract

**Background:**

Although patients with systemic lupus erythematosus (SLE) may experience various gastrointestinal disorders, SLE and Crohn’s disease (CD) rarely coexist. The diseases may have gastrointestinal (GI) manifestations, laboratory results, and radiographic findings that appear similar and consequently differentiating between GI involvement in CD and in SLE may be difficult. We present the case of a patient with SLE and CD who developed continuous GI bleeding and diarrhea that was initially treated as SLE-related colitis to little effect.

**Case presentation:**

A 55-year-old Japanese woman with systemic lupus erythematosus (SLE) developed continuous gastrointestinal bleeding and diarrhea since the patient was aged 30 years that was initially treated as SLE-related colitis. Although a longitudinal ulcer and aphthous ulcers in the colon were observed every examination, biopsy showed only mild inflammation and revealed neither granuloma nor crypt abscess. The patient underwent surgery for anal fistulas twice at 50 and 54 years of age and her symptoms were atypical of lupus enteritis. Colonoscopy was performed again when the patient was 55 years of age because we suspected she had some type of inflammatory bowel disease (IBD). Cobblestone-like inflammatory polyps and many longitudinal ulcers were detected between the descending colon and the cecum. Macroscopic examination strongly suggested CD. Histopathological examination revealed non-caseating granuloma and no evidence of vasculitis, consistent with CD. Introduction of infliximab dramatically relieved the patient’s melena and abdominal symptoms.

**Conclusion:**

Diagnostic criteria for CD and SLE overlap, making them difficult to diagnose correctly. It is important to consider CD for patients who have SLE with gastrointestinal manifestations. The pathology of lupus enteritis should be clarified through the accumulation of cases of SLE combined with CD.

## Background

Systemic lupus erythematosus (SLE) and Crohn’s disease (CD) are multisystem diseases characterized by widespread tissue damage [1]. The diseases may have gastrointestinal (GI) manifestations, laboratory results, and radiographic findings that appear similar and consequently differentiating between GI involvement in CD and in SLE may be difficult. There are, in fact, few reports suggesting an association between these diseases [2-8].

We present the case of a patient with SLE and CD who developed continuous GI bleeding and diarrhea that was initially treated as SLE-related colitis to little effect.

## Case presentation

**Figure 1:**
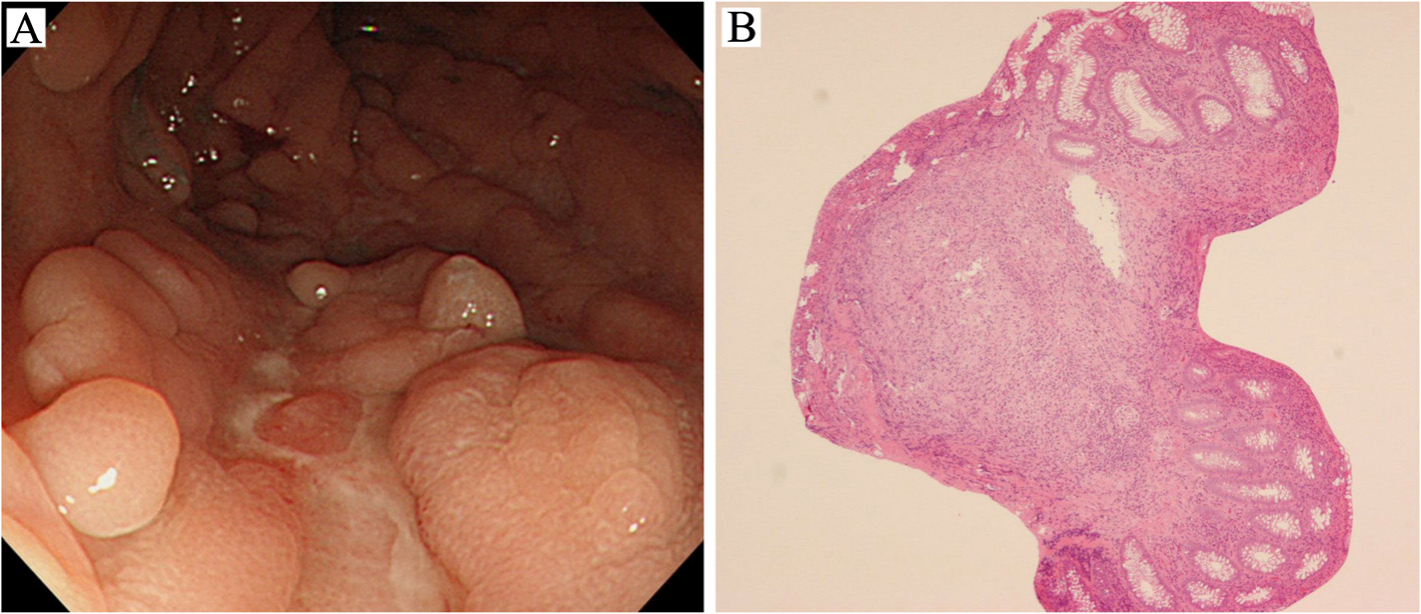
**Findings of colonoscopy and colon biopsy specimens.** (**A**) Macroscopic findings of colonoscopy with cobble-stone-like inflammatory polyps and many longitudinal ulcers in the descending colon. (**B**) Histopathological findings of specimens in A (hematoxylin and eosin, ×100) with noncaseating granuloma and no evidence of vasculitis.

A 55-year-old woman developed rash and arthralgia when she was 19 years old. Her physician at the time diagnosed her illness as SLE because of positive antinuclear antibody (ANA) and anti-double-stranded (dsDNA) antibody results and began treatment with 30 mg/day prednisolone (PSL), which was subsequently reduced to 10 mg/day. Abdominal pain accompanied by diarrhea began to occur intermittently when the patient was aged 30 years. She developed fever at the age of 31 years after swimming in the sea and was admitted to our department for treatment and close examination of her chronic diarrhea. She had a high titer of dsDNA antibody, a positive result for ANA, polyarthritis, rash, and photosensitivity; therefore, we confirmed the diagnosis of SLE using the American Rheumatism Association criteria. Although colonoscopy revealed a linear ulcer, no granuloma was detected by biopsy. Due to melena and abdominal pain, she again underwent colonoscopy when she was 32 and 33 years of age. Although a longitudinal ulcer in the descending colon (D/C) and aphthous ulcers in the transverse colon (T/C) and the ascending colon (A/C) were observed during each examination, biopsy showed only mild inflammation and revealed neither granuloma nor crypt abscess. Her PSL dose was increased to 60 mg/day during each of these hospital visits, because her symptoms were assumed to result from exacerbation of lupus enteritis. However, remission was not achieved. GI symptoms were exacerbated whenever the PSL dose was reduced to ≤15 mg/day. Colonoscopy performed because of diarrhea when the patient was 39 and 49 years old revealed longitudinal ulcers in D/C and ulcerative lesions extended over a wide area from the sigmoid colon to the cecum, no significant finding was obtained by biopsy. The patient was prescribed immunosuppressants, such as azathioprine (AZA), methotrexate (MTX), or tacrolimus (TAC) in addition to PSL 10–20 mg/day and salazosulfapyridine (SASP) 3 g/day at an outpatient unit of the hospital. However, the patient repeatedly had melena, positive for immunological fecal occult blood, and positive for inflammatory responses. The patient underwent surgery for anal fistulas twice at 50 and 54 years of age and her symptoms were atypical of lupus enteritis. Colonoscopy was performed again when the patient was 55 years of age because we suspected she had some type of inflammatory bowel disease (IBD). Cobblestone-like inflammatory polyps and many longitudinal ulcers were detected between the descending colon and the cecum (Figure 1A). Macroscopic examination strongly suggested CD. Histopathological examination revealed non-caseating granuloma and no evidence of vasculitis (Figure 1B), consistent with CD. On November 9, 2009, the patient was hospitalized to begin infliximab treatment for CD at 55 years of age. Hemoglobin (Hb) was 10.9 g/dl (normocytic), white blood cell count was 9.44 × 10^3^/ml (neutrophilic leukocytes: 90.8%, lymphocytes: 7.4%), and platelets were 4 × 10^5^/μl. Serum blood urea nitrogen (BUN) was 18.7 mg/dl and creatinine (Cre) was 0.75 mg/dl, with normal electrolytes, glucose, and liver function tests. Prothrombin time (PT), partial thromboplastin time (PTT), fibrinogen, and D-dimers were normal. C-reactive protein (CRP) and erythrocyte sedimentation rate were 1.19 mg/dl and 48 mm/h, respectively. Other laboratory tests indicated the presence of ANA, anti-SS-A antibodies, normal complement levels, and a high titer of anticardiolipin (aCL)-β2GPI (68.9 U/ml). The tests for anti-dsDNA antibodies had become negative because of the administration of steroid. Urinalysis was normal and stool cultures did not reveal any infection. Her stools were positive for occult blood.

Introduction of infliximab dramatically relieved the patient’s melena and abdominal symptoms. Infliximab was administered at a dose of 5 mg/kg body weight (340 mg) at weeks 0, 2, and 6, and then every 8 weeks thereafter. Regarding inflammatory responses after 1 month of infliximab administration, the CRP level normalized from 1.19 mg/dl to 0.06 mg/dl, and the erythrocyte sedimentation rate (ESR) from 48 mm/hr to 14 mm/hr. The Harvey-Bradshaw index (HBI) score was also reduced from 5 to 0. The patient had sustained lack of inflammatory response and negative test results for fecal occult blood. Ultimately, the PSL dose was reduced to 5 mg/day, and both MTX and TAC were tapered to discontinuation. As of May 2012, remission is maintained with infliximab, 5 mg/day PSL, and 3 g/day SASP. However, the aCL-β2GPI antibody titers remain high.

## Discussion

Although patients with SLE may experience various GI disorders, SLE and CD rarely coexist [2-8]. Table 1 lists reports of the combination of SLE and CD. Except in Case 4, CD developed a mean of 12.0±12.2 years after the onset of SLE symptoms. In the case reported here, the patient developed regular CD more than 36 years after onset of SLE. All cases were positive for anti-dsDNA antibodies or anti-DNA antibodies. Polyarthritis occurred as an SLE symptom in five of the seven cases. The frequency of peripheral arthritis among CD patients is 1–22% and symptoms similar to those of collagen disease may develop [9]. Therefore, a causal relationship between SLE and CD cannot be excluded.

**Table 1:** Comparison of reported patients with SLE complicating Crohn disease

**Pt**	**Age/Sex**	**SLE disease duration**	**Immunological findings**	**Sympotom**	**ESR (mm/hr)**	**Colonoscopy findings**	**Result of biopsy**	**Treatment**	**Reference**
1	28M	7years	ANA 1280× Anti-DNA 160×	Diarrhea	89	Deep linear and ulceration, pseudopolyps, skip lesion	Acute and clonic inflammation	mPSL40mg/d. d iv	[2]
				pyoderma gangrenosum					
2	15F	3years	ANA 1280× Anti-DNA 50×	Abdominal pain	68	Multiple ulcers with linear ulcer, skip lesion, Pseudpolyps	Infiltration of chronic inflammatory cells in the lamina proprial mucosa with marked depletion of goblet cells without vasculitis	Salazosulpha-pyridine	[3]
				Diarrhea					
				Blood stained stool					
3	55F	12years	ANA 80× Anti-dsDNA 80×positive LE cell	Intermittent hematochezia, tenesmus and loose bowel movements	35	Multiple ulcers with linear ulcer, diffuse aphthous ulcers	active colitis with noncaceating granulomas	Prednisone	[4]
4	25F	She developed SLE four years after developing Crohn’s disease.	ANA 160× Anti-dsDNA 800IU/ml pANCA positive	Watery diarrhea	N/A	Longitudinal ulcers and mucosal erosion	Focal cryptitis with noncaceating granuloma	Salazosulpha-pyridine 3g/d	[5]
				Lower abdominal pain, Perianal abscess					
5	37M	9years	ANA 320× Anti-dsDNA320× Anti-DNA 26IU/ml positive LE cell	Diarrhea	65	Longitudinal ulcers, linear ulcer, cobble stone appearance, Pseudpolyps	Non-specific colitis without vasculitis	Salazosulpha-pyridine 1g/d,Azathiop-rine75mg	[6]
				hematochezia					
6	49F	5years	ANA positive	Diarrhea	N/A	Emergent operation with	Transmural fibrosis and	Mesalazine	[7]
			anti-dsDNA 234IU/ml	Abdominal pain		a right hemicolectomy was performed.	inflammation with lymphocyte aggregation, but no evidence of vasculitis.	Prednisone 10mg b.i.d.	
				Massive bloody stool					
7	55F	36years	a high titer of anti-dsDNA antibody (at diagonosis of SLE) a positive result for ANA	Diarrhea	48	Longitudinal ulcers, cobble stone appearance, Pseudpolyps	active colitis with noncaceating granulomas without vasculitis	Infliximab	The present case
				Abdominal pain					
				bloody stool					
				anal fistula					

M,male; F, female; ANA, antinuclear antibody; Anti-DNA, antinative deoxyribonucleic acid antibody; mPSL, methylprednisolone; N/A, not available; Anti-dsDNA,antinative double-stranded antinative deoxyribonucleic acid antibody.

SLE is a multisystemic disease; therefore, patients frequently present with GI disorders that may be clinically similar to CD. However, there are some differences [4]. Compared to SLE, CD presents more frequently as diarrhea, abdominal pain, and anal lesions. All seven patients described in Table 1 experienced diarrhea, whereas five experienced abdominal pain, and two had anal lesions. CD has greater ileal involvement with a more segmented distribution than SLE, and radiographs show deep ulcers, fissures, and a cobblestone appearance or fistula formation as well as the macroscopic colonoscopy findings. Plain film radiographs of SLE-related mesenteric vasculitis usually show nonspecific indications of disease, such as segmental bowel dilatation in a thumb-print pattern and an air–fluid level. CT scans reveal the characteristic features of IBD (e.g., a double halo sign and comb-like appearance of the supplying vessels). Angiography of the mesenteric arteries may also provide evidence of vasculitis.

The most common pathological lesions in the GI tract of patients with SLE are chronic, nonspecific mucosal inflammation and ischemic changes due to vascular lesions. However, vasculitis was not confirmed in any of the cases presented in Table 1. Therefore, the abdominal symptoms experienced by patients with both SLE and CD can be attributed to CD alone.

Additionally, perforation rarely occurs in IBD, but is often observed in lupus enteritis, and may be helpful in differential diagnosis [10]. GI lesions associated with SLE are roughly classified into two types: (1) those associated with vasculitis and that frequently cause perforation; and (2) nonspecific ulcerative or granulomatous colitis [11]. The latter indicate that CD is complicating SLE. Therefore, when encountering steroid-resistant SLE-associated enteritis with the typical findings of CD, such as macroscopic findings of cobblestone-like inflammatory polyps, histopathological findings of noncaseating granuloma, and the presence of discontinuous longitudinal ulcers and aphthous ulcers in the colon, a diagnosis of CD should be considered in the differential, and anti-TNF-α therapy should be kept in mind.

Patients with IBD that is treated with sulfasalazine can, in rare cases, develop drug-induced lupus syndrome [12]. Drug-induced lupus syndrome is associated with negative ANA laboratory tests and hypocomplementemia. The patient described here was positive for ANA and anti-dsDNA antibody before the administration of SASP when she was 19 and 31 years old; therefore, drug-induced lupus syndrome was unlikely.

In early CD lesions, such as aphthous ulcers associated with noncaseating granuloma, macrophagic epithelioid cells aggregate in response to antigens from food and enteric bacteria that have invaded the intestinal mucosa. These macrophagic cells produce proinflammatory cytokines, such as interleukin-6 and tumor necrosis factor (TNF)-α, which are considered to be important for the development of CD. It is for this reason that CD is classified as an autoimmune disease [13]. In the case described here, administration of steroids in early-stage SLE might have delayed the onset of obvious lower GI CD lesions.

The overall prevalence of aCL antibodies among CD patients is approximately 22% [14]. Although the patient was strongly positive for aCL antibodies, coagulation factor levels were always within the normal range. Moreover, the patient had no history of thrombosis. Thus, concomitant antiphospholipid syndrome is unlikely.

Glucocorticosteroid is an effective treatment for CD, and can be used to treat severe cases or disease that does not respond well to mesalazine therapy. However, longterm treatment with glucocorticosteroids should be avoided. In case of steroid dependency or steroid refractory TNF-alpha blockers are an effective treatment to induce and maintain remission [15]. In the case described herein, CD activity was not controlled by high-dose PSL therapy or by moderate doses of steroids and SASP. However, a complete response was achieved with anti-TNF-α therapy. TNF-α inhibitors have been reported to cause drug-induced lupus, as well as rashes and arthritis [16]. Fortunately, in this case, SLE symptoms did not worsen. There are, however, occasional reports describing the efficacy of anti-TNF-α therapy for SLE. It has also been reported that, despite levels of antibodies to ds-DNA and cardiolipin being increased, anti-TNF-α therapy did not exacerbate SLE itself but rather achieved a reduction in disease activity and relief of refractory arthritis, nephritis, etc. [17]. TNF-α exerts both deleterious tissue damaging effects mainly through its pro-inflammatory activities and beneficial activities by dampening aggressive autoimmune responses. SLE is a disease with autoimmune disturbance and inflammatory damage, so blocking TNF-α in this autoimmune-prone chronic inflammatory disease may lead to different outcomes, depending on timing and duration of treatment[18]. Thus, infliximab may also be effective for gastrointestinal symptoms associated with SLE.

## Conclusion

In conclusion, the diagnostic criteria for CD and SLE overlap, making them difficult to diagnose correctly. It is important to consider the possibility of CD in patients who have SLE with GI manifestations. The status of CD as an autoimmune disease is becoming clear and the pathology of lupus enteritis should be clarified through the accumulation of cases of SLE combined with CD.

## Consent

Written informed consent was obtained from the patient for publication of this Case report and any accompanying images. A copy of the written consent is available for review by the Series Editor of this journal.

## Competing interests

None of the authors including Hiroyuki Yamashita, Yo Ueda, Hishimi Kawaguchi, Akitake Suzuki, Yuko Takahashi, Toshikazu Kano and Akio Mimori have any conflicts of interest associated with this case report.

Financial competing interests.

In the past five years have you received reimbursements, fees, funding, or salary from an organization that may in any way gain or lose financially from the publication of this manuscript, either now or in the future? Is such an organization financing this manuscript (including the article-processing charge)? If so, please specify. No.

Do you hold any stocks or shares in an organization that may in any way gain or lose financially from the publication of this manuscript, either now or in the future? If so, please specify. No.

Do you hold or are you currently applying for any patents relating to the content of the manuscript? Have you received reimbursements, fees, funding, or salary from an organization that holds or has applied for patents relating to the content of the manuscript? If so, please specify. No.

Do you have any other financial competing interests? If so, please specify. No.

Non-financial competing interests.

Are there any non-financial competing interests (political, personal, religious, ideological, academic, intellectual, commercial or any other) to declare in relation to this manuscript? If so, please specify. No.

## Authors’ contributions

This manuscript was written under the role assignment specified below. I, HY, serving as the lead and corresponding author, was primarily in charge of manuscript drafting and data analysis. YU, HK, AS, YT, TK, members of the Division of Rheumatic Diseases, National Center for Global Health and Medicine in charge of assessing the disease history of a patients. AM (Director of our institute’s Division of Rheumatic Diseases) supervised drafting of the manuscript and gave final approval of the version to be published. All authors read and approved the final manuscript.

## Authors’ information

All of authors, H.Yamashita,MD,PhD;Y.Ueda,MD;H.Kawaguchi,MD;A.Suzuki,Md,PhD; Y.Takahashi,MD,PhD;T.Kano,MD; A. Mimori,MD,PhD, belong to the Division of Rheumatic Diseases ,National Center for Global Health and Medicine,Shinjuku-ku, Tokyo-to, Japan.

## Pre-publication history

The pre-publication history for this paper can be accessed here:

http://www.biomedcentral.com/1471-230X/12/174/prepub
